# Implications of Dengue Virus Maturation on Vaccine Induced Humoral Immunity in Mice

**DOI:** 10.3390/v13091843

**Published:** 2021-09-15

**Authors:** Connor A. P. Scott, Alberto A. Amarilla, Summa Bibby, Natalee D. Newton, Roy A. Hall, Jody Hobson-Peters, David A. Muller, Keith J. Chappell, Paul R. Young, Naphak Modhiran, Daniel Watterson

**Affiliations:** 1 School of Chemistry and Molecular Biosciences, The University of Queensland, St. Lucia, QLD 4072, Australia; Connor.scott@uq.net.au (C.A.P.S.); a.amarillaortiz@uq.edu.au (A.A.A.); summa.bibby@uq.net.au (S.B.); natalee.newton@uq.edu.au (N.D.N.); roy.hall@uq.edu.au (R.A.H.); j.peters2@uq.edu.au (J.H.-P.); d.muller4@uq.edu.au (D.A.M.); k.chappell@uq.edu.au (K.J.C.); p.young@uq.edu.au (P.R.Y.); 2 Australian Infectious Diseases Research Centre, The University of Queensland, St. Lucia, QLD 4072, Australia

**Keywords:** dengue, vaccination, ADE, virology, microbiology, flavivirus

## Abstract

The use of dengue virus (DENV) vaccines has been hindered by the complexities of antibody dependent enhancement (ADE). Current late-stage vaccine candidates utilize attenuated and chimeric DENVs that produce particles of varying maturities. Antibodies that are elicited by preferentially exposed epitopes on immature virions have been linked to increased ADE. We aimed to further understand the humoral immunity promoted by DENV particles of varying maturities in an AG129 mouse model using a chimeric insect specific vaccine candidate, bDENV-2. We immunized mice with mature, partially mature, and immature bDENV-2 and found that immunization with partially mature bDENV-2 produced more robust and cross-neutralizing immune responses than immunization with immature or mature bDENV-2. Upon challenge with mouse adapted DENV-2 (D220), we observed 80% protection for mature bDENV-2 vaccinated mice and 100% for immature and partially mature vaccinated mice, suggesting that protection to homotypic challenge is not dependent on maturation. Finally, we found reduced in vitro ADE at subneutralising serum concentrations for mice immunized with mature bDENV-2. These results suggest that both immature and mature DENV particles play a role in homotypic protection; however, the increased risk of in vitro ADE from immature particles indicates potential safety benefits from mature DENV-based vaccines.

## 1. Introduction

DENV, a member of the Flaviviridae, has four distinct serotypes (1–4) and is transmitted to vertebrate hosts by *Aedes* mosquitos. It poses a substantial risk to public health with an estimated 390 million cases each year [1]. While often causing a self-limiting febrile illness, known as dengue fever, it can also result in severe dengue disease, which is characterized by dengue hemorrhagic fever and dengue shock syndrome (DHF/DSS) [2,3]. Following infection with one serotype, humans develop both lifelong type-specific and transient cross serotype immunity [3,4]. Severe dengue disease is associated with secondary anamnestic dengue infection, which is likely explained in part by antibody dependent enhancement (ADE) [5]. ADE occurs when the antibodies developed from primary infection increase uptake of a secondary heterosubtypic virus on the Fc receptor bearing cells such as macrophages (extrinsic ADE) and modulate innate and adaptive immune responses (intrinsic ADE) to enhance infection [6]. Reducing the risk of ADE requires a vaccine/therapeutic strategy that provides broad and potent protection from all four serotypes.

The DENV genome is composed of a single open reading frame flanked by 5′ and 3′ noncoding regions that is post-translationally processed by viral and host proteases into three structural (capsid, C; pre-membrane, prM; envelope, E) and seven non-structural (NS1–NS5) proteins. The E protein is the major surface antigen on the mature virion and is composed of three domains: EDI, EDII, EDIII, and a transmembrane domain. Newly assembled virions bud from the endoplasmic reticulum (ER) membrane, acquiring 180 copies of the E and prM proteins on their surface. The non-infectious immature virion that buds from the ER membrane has a spiky surface composed of tripod-like trimers of prM-E heterodimers [7,8,9]. The low-pH environment of the trans-Golgi network results in the dissociation of the trimeric prM-E heterodimers, which collapse into a smooth low pH intermediate. This conformational change exposes the cleavage site present on prM and allows host furin-like proteases to cleave pr from M. The resulting virion egressed from the cell is considered the mature infectious particle comprising of 90 E-M dimers. 

The maturation of DENV is inefficient, with up to 40% of virus particles in cell culture being partially or fully immature [10]. This is mediated by a highly conserved acidic residue at position 3 (P3) of the DENV pr-M junction, which reduces prM cleavage by furin [10,11]. By comparison, other flaviviruses, such as West Nile virus (WNV) and Zika virus, have polar serine residues at P3, making their furin cleavage sites more efficiently processed [12]. Previous efforts to optimize DENV maturation have been achieved through the mutagenesis of the P3 residue and growth in furin overexpressing cell lines [11,12,13]. Although immature vertebrate-infecting flavivirus particles are non-infectious, they become highly infectious in the presence of prM antibodies [14,15]. These antibodies dominate the human antibody response to DENV infection and are both highly cross-reactive and weakly neutralizing across serotypes [16]. 

In the mature virion, the cleaved M protein acts as a pH sensitive latch, preventing the premature exposure of the fusion loop and incorrect fusion [17]. The conserved linear fusion loop epitope (FLE) is readily exposed on the immature structure and is poorly presented on the mature virion [18]. The mature virion undergoes dynamic E dimer ‘breathing’, a process in which the tip of EDII hinges at the EDI–EDII interface at biological temperatures, causing increased fusion loop exposure [19,20,21]. Antibodies to the fusion loop epitope are often weakly cross-neutralizing and are broadly cross-reactive [22,23]. Potent neutralizing and serotype-specific human antibodies often target the lateral ridge of EDIII, the hinge region of EDI/II, and/or the E dimer epitope (EDE). EDE antibodies potently neutralize all DENV serotypes and show reduced in vitro ADE when compared to FLE antibodies [24]. Antibodies to these regions preferentially bind and neutralize the mature virions and the mature components of partially mature virions rather than immature virions [24,25]. 

Currently there are three live-attenuated vaccines in Phase III trials or with restricted licensure: CYD-TDV, TV003/TV005, and DENVax [26,27,28,29]. Beyond these, there are several inactivated, subunit, and DNA vaccines in Phase I safety trials [30,31,32]. The extent of virus maturation on inactivated and live-attenuated vaccines is largely unclear. CYD-TDV has been reported to produce particles of heterogenous maturity [33], and it is likely that other virus-based vaccines that utilize native DENV prM cleavage sites would behave similarly. Cell culture propagated DENV-1 has recently been shown to produce partially mature particles, while the genetically identical virus that circulates in humans produces highly mature particles [34]. The effects of particle maturation status in DENV vaccine efficiency remains to be rationally explored. As such, we utilized a recently reported chimeric flavivirus vaccine candidate, bDENV-2 [35], based off the insect specific Binjari (BinJV) virus [36], to investigate the differential humoral immunity generated in AG129 mice to mature, immature, and partially mature DENV particles. More specifically, this will assist in further understanding the role of immature and partially mature virions in ADE and protection from DENV. 

## 2. Materials and Methods

### 2.1. Animal Ethics

Animal experiments were approved by the University of Queensland Animal Ethics Committee (AEC nos. SCMB/010/019 and SCMB/011/019) and were performed in accordance with National Health and Medical Research council guidelines. Animals were housed under specific pathogen-free conditions in the UQBR animal housing facility at the Australian Institute for Bioengineering and Nanotechnology. 

### 2.2. Cell Culture

C6/36 (*Aedes albopictus)* cells were maintained at 28 °C in Rosewell Park Memorial Institute (RPMI) 1640 media supplemented with 5% heat-inactivated fetal bovine serum (FBS). Vero (African green monkey kidney) cells were maintained at 37 °C with 5% CO_2_ in Opti-MEM reduced-serum media supplemented with 5% FBS. K562 cells were maintained at 37 °C with 5% CO_2_ in RPMI 1640 supplemented with 10% FBS. Both the RPMI 1640 and Opti-MEM media were also supplemented with 100 U/mL of penicillin, 100 μg/mL of streptomycin, and 2 mmol/L of L-glutamine.

### 2.3. Virus Generation

The bDENV-2 chimeric viruses were generated using a circular polymerase extension reaction, as previously described [37,38]. The RNA of BinJV (Accession: MG587038.1) and DENV-2 (ET-300 strain, Accession: EF440433) was extracted using Trizol LS reagent (ThermoFisher Scientific, Waltham, MA, USA) and was converted to cDNA using Superscript III RT (Thermo Fisher). The prM and E genes of DENV-2 and the 5 non-structural genes, C and the UTRs of BinJV, were amplified with complementary overhangs for the subsequent gene. DENV-2 prME cleavage was optimized using two overlapping mutagenesis PCR products, as previously described [39]. Each fragment was purified and combined in equimolar concentrations (0.1 pmol) to create a GXL PCR reaction (TakaraBio, Shiga, Japan) and was incubated as previously described [38]. The reaction was then transfected into C6/36 cells using Effectene (Qiagen, Hilden, Germany) as per the manufacturer’s guidelines. Successful virus recovery was tested by immunofluorescence assay (Figure S1A), and prME gene sequences were confirmed using sanger sequencing (Figure S1B). Furin optimized bDENV-2 will herein be termed bDENV-2_F-Opt_.

### 2.4. Virus Propagation and Purification

C6/36 cells at ~90% confluency were infected with bDENV-2 or bDENV-2_F-Opt_ at a multiplicity of infection (MOI) of 0.001. The supernatant was harvested on days 3, 5, and 7 post infection and were clarified by centrifugation at 4000× *g* for 10 min at 4 °C before filtration through a 0.22 µm filter (Merck Millipore, Burlington, MA, USA). The media was replenished after each harvest with RPMI containing 2% FCS. To propagate immature viruses, the cells were infected as described above with bDENV-2. On day 2 post infection, the media were removed and were replaced with RPMI supplemented with 2% FCS and 20 mM NH_4_Cl. Supernatant was harvested on days 5 and 7 post infection. The virus supernatant was combined with 8% polyethylene glycol (PEG) 8000 and precipitated overnight on a magnetic stirrer before centrifugation at 11,899× *g* for 1 h at 4°. The virus pellet was suspended in cold NTE buffer (12 mM Tris, 120 mM NaCl, and 1 mM EDTA (pH 8)) prior to ultracentrifugation through a 20% sucrose cushion and 25–40% potassium tartrate gradients, as previously described [39,40]. The purified virus was harvested, and buffer exchanged into NTE and concentrated using a 30 kDa Amicon filter (Merck Millipore, Burlington, MA, USA). Virus purity was assessed on 4–15% Mini-PROTEAN TGX precast protein gels (Bio-Rad, Hercules, CA, USA) and was stained with Coomassie Blue. The gels were destained (40% Methanol, 10% Acetic acid) for 2–3 h until the protein bands were visible. Virus stocks of DENV-1 (ET-243, Accession: EF440432), DENV-2 (ET-300), DENV-3 (ET-209, Accession: EF440434), and DENV-4 (ET-288, Accession: EF440435) were propagated in C6/36 cells and were titrated on Vero cells.

### 2.5. Recombaint Monoclonal Antibody Expression and Purification

Mammalian expression plasmids [41] encoding the heavy and light chains of mAbs 4G2 [42], C8 [24], 513 [43], and DV62.5 [22] were transfected into ExpiCHO-S (ThermoFisher Scientific, Waltham, MA, USA) cells as per the manufacturer’s instructions and were incubated on an orbital shaker at 120 rpm with 7.5% CO_2_ for seven days at 37 °C. The supernatant was harvested from the cells via centrifugation at 4800× *g* for 30 min, filtered through 0.22 μm filters (Merck Millipore, Burlington, MA, USA), and purified using AKTA-FPLC affinity chromatography with a 1 mL HiTrap Protein A HP column (GE Healthcare, Chicago, IL, USA). Eluate was buffer exchanged into PBS and was concentrated using 30 kDa molecular weight cut-off centrifugal filter units (Merck Amicon, Burlington, MA, USA).

### 2.6. Immunisation of AG129 Mice with bDENV-2

Female AG129 mice (18–21 weeks old) were divided into four groups of five mice and were immunized intradermally with 20 μL of PBS (Group 1), immature bDENV-2 (Group 2), partially mature bDENV-2 (Group 3), and mature bDENV-2_F-Opt_ (Group 4), all of them were adjuvanted with Quil-A (QA) under anesthetic. Each mouse received a 1 μg dose of virus, which was quantified by the amount of E protein in each virus preparation using SDS-PAGE separated BSA standards, and 3 μg of QA. The mice received a booster dose 21 days after their first immunization. Tail bleeds were taken on days 20 and 34 post first immunization. Serum was recovered from the tail bleeds by centrifugation at 10,000× *g* for 20 min at 4 °C and was stored at −20 °C until needed for analysis. 

### 2.7. Virus Challenge

The mice were challenged 27 days after their second immunization with 5 × 10^5^ plaque forming units (pfu) of the mouse adapted DENV-2 D220 [44] via intraperitoneal injection (i.p.) (kindly provided by Prof. Eva Harris, University of California, Berkeley). Daily bleeds were taken for 7 days post infection (dpi). Sera were collected as described above. Clinical scores and weight loss for the mice were observed and recorded for 14 days post infection [45]. 

### 2.8. ELISAs

Ninety-six well Maxisorp ELISA plates (ThermoFisherScientific, Waltham, MA, USA) were coated with 2 µg/mL of bDENV-2 in 50 µL of PBS and were incubated at 4 °C overnight. The plates were then blocked with 150 µL of blocking buffer (5% milk diluent blocking concentrate (KPL) in PBS with 0.05% Tween 20 (PBS-T)) for 1 h at room temperature. The buffer was discarded, and 50 µL of serially titrated primary antibody in blocking buffer was added to each well. The plates were incubated at 37 °C for 1 h before being washed three times by means of H_2_O emersion and were dried. The HRP-conjugated secondary antibody (Sigma-Aldrich,. St. Louis. MO, USA) (1:1000 dilution in blocking buffer) was added to each well. The plates were incubated at 37 °C for 1 h before being washed as above. Antibody binding was revealed by applying 50 µL of tetramethylbenzidine (TMB) (Life Technologies, Carlsbad, CA, USA) into each well, incubating 5 min, and then applying 25 µL of 1 M sulphuric acid. The OD was measured at 450 nm using a Varioskan LUX Microplate Reader (ThermoFisher Scientific, Waltham, MA, USA). The dissociation constant (K_d_) values were then determined using a one-site specific binding non-linear fit model on GraphPad Prism 9 software.

### 2.9. Plaque Reduction Neutralisation Tests (PRNTs)

Vero cells were seeded at a density of 4 × 10^4^ cells/well in a 96-well plate (Thermo Fisher) and were incubated overnight at 37 °C with 5% CO_2_. Mouse sera obtained after the first and second immunizations were heat inactivated at 56 °C for 30 min. Individual and pooled sera were diluted 1:25 and 1:50, respectively, and were 2-fold serially diluted in serum free Opti-MEM using a 96-well round bottom plate. An equal volume containing 100–150 FFU of DENV-1, -2, -3, or -4, was added to each well and was incubated for 1 h at 37 °C with 5% CO_2_. The sera–virus complexes were transferred to Vero cells and were incubated for 1 h at 37 °C. Following incubation, 150 μL of overlay medium was added to the cells, which were then incubated at 37 °C with 5% CO_2_ for 48 h. The overlay media contained 1.5% carboxymethlycellulose in M199 media (ThermoFisher Scientific, Waltham, MA, USA) supplemented with 2% FCS, 100 U/mL penicillin, and 100 μg/mL streptomycin. After incubation, the media were removed, and the cells were fixed with ice cold 80% acetone in PBS for 30 min at −20 °C. The immunoplaques were stained as previously described [45]. In brief, the plates were blocked with 1× KPL in PBS-T for 1 h followed by probing with anti-E mAb 4G2 on a human IgG1 backbone [46] at 1 μg/mL for 1 h at 37 °C. The plates were washed three times with PBS-T before adding IR800 fluorophore conjugated secondary antibody (1:2500) and were incubated for 1 h at 37 °C (Millennium Science, Mulgrave, VIC, Australia). The plates were scanned using the Odyssey CLx Infrared Imaging system (LI-COR Biosciences, Lincoln, NE, USA) at a resolution of 42 μm in the 800 nm channel. The plaques were counted by eye and a 50% inhibitory concentration (IC_50_) was determined using GraphPad Prism 9 with a three-parameter (inhibitor) vs. response model.

### 2.10. Quantification of Viraemia by Immunoplaque Assay

Levels of circulating virus in the serum were determined by viral immunoplaque assay in the C6/36 cells as previously described [39]. Briefly, sera obtained from daily post infection tail bleeds were diluted in a ratio of 1:10 in serum free RPMI and were serially titrated in four 10-fold dilutions onto confluent C6/36 cells in 96-well plates and were incubated for 2 h at 28 °C with 5% CO_2_. After incubation, 150 μL of overlay media (described as above) was added to the cells and incubated at 28 °C with 5% CO_2_ for 72 h. Immunoplaque staining was performed as above.

### 2.11. NS1 Quantitative Capture ELISA

The amount of circulating NS1 antigen in the mice sera was quantified using a sandwich ELISA protocol as previously described [47]. Briefly, 96-well plates were coated overnight at 4 °C with 50 μL of capture antibody (anti-NS1 mAbs: mouse Gus11 (DENV-4), human 2A5 (DENV-1 and 2), and mouse 1G5.3 (DENV-3)) at 2 μg/mL before being blocked for 1 h at room temperature. Sera were diluted into the blocking buffer, added to the plate, and incubated for 1 h at 37 °C. The plates were washed three times in PBS and 0.05% Tween-20. The captured NS1 was probed for use as either anti-NS1 mAbs human 4G4 DENV-4 and DENV-2, mouse Gus11 (DENV-1) (2 μg/mL), or anti-NS1-HRP (DENV-3) (Panbio) and was incubated for 1 h at 37 °C. Horse radish peroxidase-conjugated goat anti-human at 2 μg/mL was added to the plates for DENV-1, -2, and -4 and was incubated for 1 h at 37 °C. Following three washes with PBS-T, the signals were developed using TMB and were measured as outlined above. Purified native NS1 from the DENV-2 (ET-300) infected cells was used as a standard to allow the quantification of the sNS1 from the infected mice sera. 

### 2.12. Antibody Dependent Enhancment Assay

ADE assays were performed as per established protocols [46]. In brief, 50 μL of mice sera (serially diluted) were incubated with equal volumes of virus at 37 °C for 1 h at a MOI of 0.05 in a v-bottom plate. Anti-E mAb 4G2 was used as a positive ADE control (Figure S2). Approximately 5 × 10^4^ cells per well in 100 μL of serum-free RPMI 1640 medium were mixed with the serum–virus complex and were incubated at 37 °C for 2 h. The cells were spun down at 500× *g* for 5 min and were washed with RPMI 1640 containing 2% FCS to remove the unbound virus and sera. The cells were then incubated for 96 h at 37 °C in a flat-bottom cell culture plate. The supernatant was harvested, and the quantity of the NS1 protein was determined using the NS1 capture ELISA outlined above. Infection enhancement was calculated by dividing the OD450 nm values in the presence and absence of sera/antibody.

### 2.13. Statistical Analysis

All statistical analysis was performed using GraphPad Prism 9. One way analysis of variance (ANOVA) was performed using Tukey’s multiple comparison with the alpha level set at 0.05. A Mantel–Cox test was performed for the statistical analysis of the survival curves.

## 3. Results

### 3.1. Generation of Mature, Immature and Partially Mature bDENV-2

**Figure 1:**
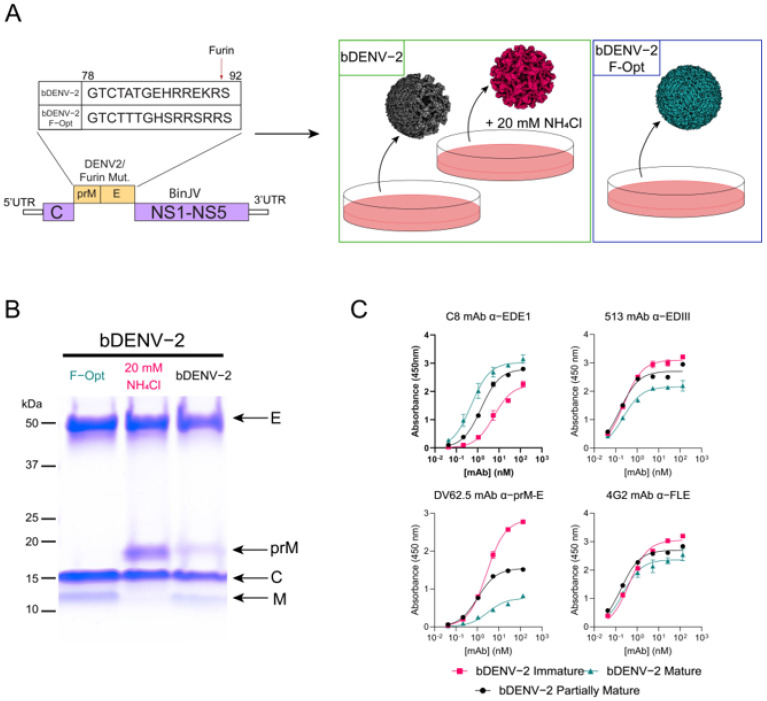
Generation of chimeric bDENV-2 viruses, analysis of purity, and mAb binding kinetics. (**A**) Schematic of chimeric bDENV-2 genome with and without pr-M cleavage site mutations and the generation of viruses of varying maturities in C6/36 cell culture. (**B**) SDS-PAGE analysis of purified virions with molecular weight ladder and structural proteins (envelope (E), pre-membrane (prM), capsid (C), and membrane (M)) indicated. (**C**) ELISA curves of prM/E specific mAbs to mature (teal), immature (pink), and partially mature (black) bDENV-2. Dissociation constants and Bmax were determined using a one-site specific binding model on Graphpad Prism 9.

To obtain mature, immature, and partially mature chimeric bDENV-2 virions, three propagation strategies were used. The mature virions were obtained by exchanging the native furin cleavage site with that of WNV (Figure 1A, left). The immature and partially mature virions were propagated using the native bDENV-2 cleavage site with and without the addition of 20 mM NH_4_Cl, respectively (Figure 1A, right). All three virus preparations were highly pure and showed distinct E (50 kDa) and C (15 kDa) proteins (Figure 1B). As previously reported [39], the modification of the prM furin cleavage site of bDENV-2 to that of WNV enhanced the furin cleavage of prM, with no detectable prM (~18 kDa) observed (Figure 1B). Immature virus particles were seen for virus propagated in the presence of NH_4_Cl, with minimal M (12 kDa) observed (Figure 1B). Antigenic differences between the viruses were characterized using indirect ELISA with a mAbs panel targeting multiple epitopes on the prM and E proteins: DV62.5 (α-prME), C8 (α-EDE1), 4G2 (α-FLE), and 513 (α-EDIII). Mature bDENV-2 showed enhanced binding kinetics to C8 when compared to the partially mature and immature viruses, confirming previous findings with other EDE mAbs [25] (Figure 1C). Reduced DV62.5 binding was also observed for mature particles compared to the immature and partially mature particles, with lower Bmax and K_d_ values being observed (Table S1), suggesting lower mAb occupancy on the virus. All three virus preparations displayed high affinity to 4G2 and 513, with comparable affinities for all three virus preparations (Figure 1C and Table S1).

### 3.2. Humoral Immune Responses to Immunisation with bDENV-2

**Figure 2:**
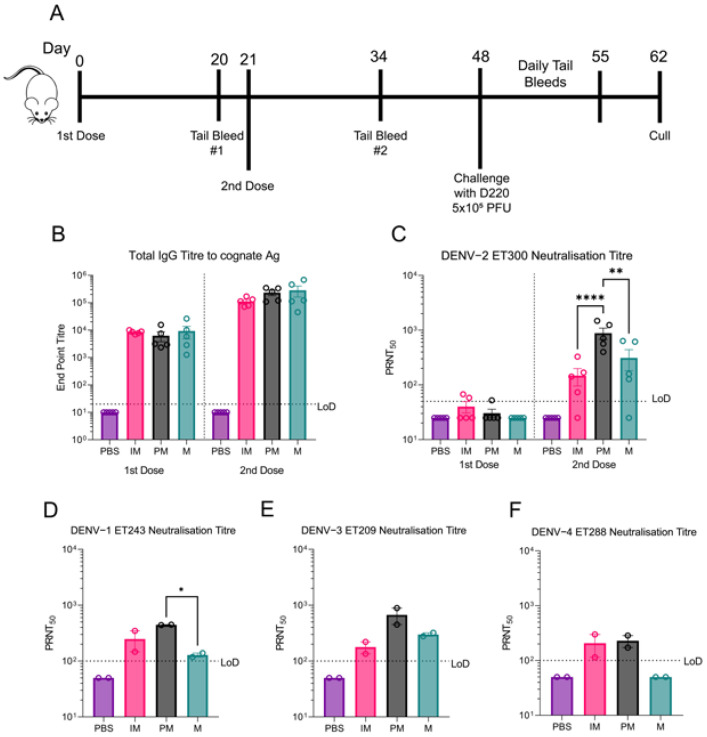
Immunogenicity of bDENV-2 in AG129 mice. (**A**) Vaccination and challenge schedule of AG129 mice with bDENV-2 and DENV-2 D220, respectively. (**B**) Total IgG titres to cognate antigen (Ag) from tail bleeds after one and two doses of bDENV-2 vaccination, as determined by indirect ELISA. End-point titre of mouse sera to cognate antigen corresponding to the reciprocal of the highest dilution that has an O.D above the mean + three standard deviations of the no sera wells on each plate. (**C**) PRNT_50_ titre of individual mouse serum against DENV-2 ET300 with error bars displaying the standard error of the mean (SEM). Neutralizing antibody titre (PRNT_50_) of pooled mice sera against DENV-1 ET243 (**D**), DENV3 ET209 (**E**), and DENV-4 ET288 (**F**). Each symbol represents a technical replicate (*n* = 2) of pooled mice sera with error bars displaying the SEM. Dotted line indicates lower limit of detection (LoD). Significance was determined using an ordinary one-way ANOVA with Tukey’s multiple comparisons test on GraphPad Prism 9.0. **** *p* < 0.0001, ** *p* ≤ 0.002, * *p* ≤ 0.03, ns *p* ≥ 0.05.

To examine the immune responses of bDENV-2 particles of varying maturities, five AG129 mice per group were immunized twice, three weeks apart, with 1 μg of mature, partially mature, or immature bDENV-2 + 3 μg of QA adjuvant (Figure 2A). The effects of the saponin-based adjuvant QA, which has been used to enhance the neutralizing immune response generated by bDENV-2 [35] and other dengue vaccines [48], on the virion structure was not directly investigated. However, the inclusion of QA within all vaccination groups equalized the effects of QA to allow direct comparison. Serum was collected after each immunization, and total antibody responses were determined using indirect ELISA against the cognate antigen (Figure 2B). No significant differences in the total IgG antibody responses were observed between the total IgG antibody responses after one or two doses, suggesting similar immunogenicity for all three virus preparations. To further analyze the immune response, the neutralization ability of the sera was tested against all four DENV serotypes using PRNT assays (Figure 2C–F). The sera from the mice immunized with partially mature bDENV-2 had significantly higher neutralization potency against DENV-2 compared to the immature (*p* < 0.0001) and mature (*p* = 0.0017) sera. A similar trend was observed for DENV-1, 3, and 4, but there were no significant differences in neutralization titres observed between the groups against DENV-3 and DENV-4 (cross-serotype PRNTs performed in duplicate using pooled sera due to availability). Surprisingly, sera from mature bDENV-2 immunized mice had significantly lower neutralization DENV-1 titres compared to the partially mature group (*p* = 0.0387), and there was no detectable DENV-4 neutralization.

### 3.3. All Maturities of bDENV-2 Provide Protection in AG129 Mice

**Figure 3:**
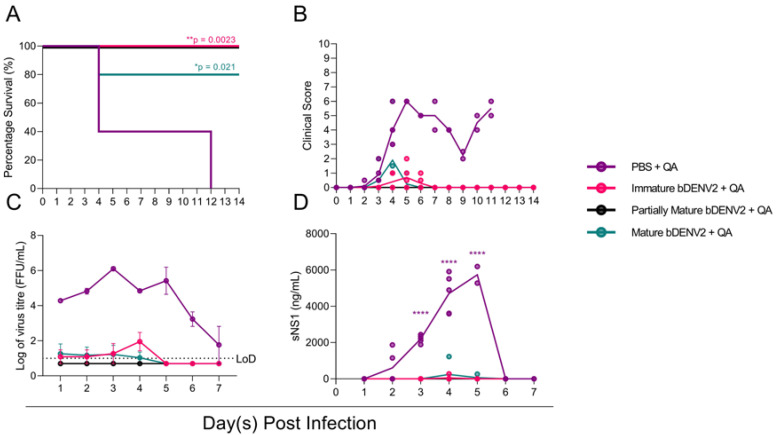
Survival, clinical score, viremia, and sNS1 levels in AG129 mice following DENV-2 D220 challenge. (**A**) Survival curve of mice immunized with bDENV-2 or PBS and challenged with 5 × 10^5^ FFU of DENV-2 D220 via i.p. injection. (**B**) Mice were observed for 14 dpi and were scored based on their locomotion, appearance, and behavior; each dot represents scores for the individual mice. Viremia (**C**) and sNS1 (**D**) were quantified by immunoplaque assay and NS1-capture ELISA. Virus titres of each mouse were determined and displayed as the mean ± SEM. Dotted horizontal line indicates a lower limit of detection (LoD). Statistical significance was determined using a log-rank (Mantel–Cox) test for survival compared to PBS + QA vaccination. The log10-transformed virus tires and sNS1 levels were analyzed using one-way ANOVA with Tukey’s multiple comparisons test. **** *p* < 0.0001, ** *p* ≤ 0.002, * *p* ≤ 0.03, ns *p* ≥ 0.05.

Upon the confirmation of a robust α-DENV immune response, the protective efficacy of the three vaccine preparations was evaluated by challenging the AG129 mice with 5 × 10^5^ pfu of DENV-2 D220. Once infected, the mice were monitored for clinical signs for 14 dpi and were bled via the tail vein for every day 7 days. The mice immunized with PBS + QA deteriorated rapidly upon infection, exhibiting clinical signs that correlated with significantly higher viral loads and NS1 levels compared to the vaccinated groups. Three mice were culled on day 4 post infection, while the remaining mice showed initial recovery before regression and were finally culled on day 12 (Figure 3A). This initial observed recovery correlated with a reduction in viremia at 6 dpi (Figure 3C). In contrast, mice immunized with bDENV-2 of all maturities showed significantly improved survival: 80% for mice immunized with mature bDENV-2 (* *p* = 0.021) and 100% for immature and partially mature/immature virions (** *p* = 0.0023) (Figure 3A). One mouse immunized with mature bDENV-2 particles was culled on day 4 post infection. This individual mouse generated a 6.3-fold lower IgG response to bDENV-2 and showed no neutralization to DENV-2 in the PRNT assays (Figure 2B,C). No viral breakthrough or sNS1 antigenemia were seen for the mice immunized with partially mature bDENV-2, while minimal viremia and sNS1 were observed for mice immunized with mature bDENV-2 and immature bDENV-2 (Figure 3 C,D). These results correlated with lower clinical scores for all of the vaccinated groups compared to the PBS control group. 

### 3.4. In Vitro Enhancment of DENV by bDENV-2 Immune Sera

**Figure 4:**
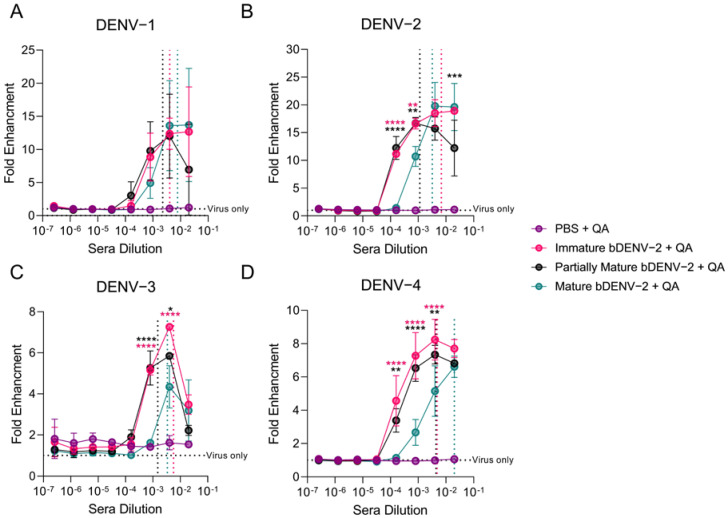
ADE of DENV 1–4 by AG129 immune sera. Fold enhancement of infection by AG129 immune sera in K562 cells relative to virus only control of DENV-1 (**A**), DENV-2 (**B**), DENV-3, (**C**), and DENV-4 (**D**), as determined by sNS1 capture ELISA. Dotted vertical lines depict the mean PRNT_50_ values of AG129 immune sera to each virus. Each data point represents the mean of two independent replicates ± SD. Significance was determined using an ordinary one-way ANOVA with Tukey’s multiple comparisons test on GraphPad Prism 9.0. **** *p* < 0.0001, *** *p* < 0.0002, ** *p* ≤ 0.002, * *p* ≤ 0.03, ns *p* ≥ 0.05.

With no substantial differences in protective efficacy observed between the three vaccination groups, we analysed the ability of the murine immune sera to induce in vitro ADE in a FcγII-receptor bearing K562 cells. K562 cells are weakly permissive to DENV infection, allowing antibody mediated entry through Fc receptors to be examined [31,47]. In this assay sNS1 levels in the cell supernatant were used as a correlate of enhancement and were evaluated by capture ELISA. No infection enhancement by PBS + QA immunized mice sera was observed for all four serotypes (Figure 4A–D). By contrast, enhancement of DENV 1–4 was observed from all of the bDENV-2 immunized mice sera from 1:50–1:6250 dilutions of sera (Figure 4A–D). The robust cross-neutralizing antibody response of the partially mature bDENV-2 immunized mice sera correlated with the peak enhancement titres in the in vitro ADE assay (DENV-1, -3, -4: 1:250, DENV-2: 1:1250). By contrast, peak enhancement was observed at lower dilutions (between 1:50 and 1:250) for all of the serotypes with the immature and mature bDENV-2 sera, which also correlated with lower mean PRNT_50_ titres. Interestingly, the mature bDENV-2 sera showed significantly reduced enhancement at higher serum dilutions (1:1250–1:6250) of DENV-2–4 when compared to immature and partially mature bDENV-2 sera (Figure 4B,D). This trend was observed despite there being no significant difference in the a-DENV-2 IgG titres to the cognate vaccine antigen between the groups (Figure 2B). As expected, α-FLE mAb 4G2 enhanced the infection of all four DENV serotypes (Figure S2).

## 4. Discussion

The search for a safe and highly efficacious vaccine for DENV remains elusive, with restrictions limiting the use of the only approved vaccine, CYD-TVD, due to safety concerns in naïve individuals [49]. In this study, we utilized a chimeric insect specific virus vaccine candidate, bDENV-2, to analyze the murine humoral immune responses to mature, partially mature, and immature DENV particles. We showed that immunization with mature, immature, or partially mature bDENV-2 resulted in robust IgG responses to DENV-2 and protection from DENV-2 D220 challenge in an AG129 model. Further, we showed that the virion maturation state plays a role in neutralizing antibody generation, with partially mature bDENV-2 inducing higher serum titres of cross-neutralizing antibodies. Finally, we showed that immunization with mature bDENV-2 reduced the in vitro ADE of DENV at subneutralising serum titres.

There is growing evidence to suggest that the most potent neutralizing antibodies for DENV target epitopes present on the mature virion [24,25]. In contrast, we observed that immunization with partially mature bDENV-2 adjuvanted with QA resulted in higher neutralization titres for all four serotypes despite having similar total IgG titres. Cross-reactive antibodies to prM and the FLE dominate the human humoral immune response to DENV infection, with serotype protective type-specific antibodies only making up a small portion of the antibody response [50,51]. This dominant anti-prM response is a result of the well-established inefficient furin cleavage of prM during DENV maturation [10,11,13]. To overcome this inefficient furin cleavage, we engineered bDENV-2 viruses with an efficient furin cleavage site at the pr-M junction by transposing the cleavage site of WNV into bDENV-2. The enhanced cross-neutralization observed from immunisation with partially mature bDENV-2 can potentially be explained by the highly conserved and cross-reactive epitopes readily presented on the immature structure of the partially mature virion [16,18,22]. The use of the adjuvant QA may impact the virion structure, but as it was included in all preparations, the effect is expected to be equalized. It is also possible that the enhanced neutralization we observed may be attributed to partially mature bDENV-2 immunisation mimicking the native morphology of the clinical isolates used in the neutralization assays where both mature and immature virion morphology is present. Rajendra et al. [34] demonstrated that cell culture derived DENV-1 was efficiently neutralized by cross-reactive fusion loop mAbs and postulated that partially mature virions would likely overestimate the efficacy of vaccines producing heterotypic neutralizing antibodies. Similar observations have been made in other flavivirus immunisation studies, whereby human serum from prME based WNV vaccines showed the preferential neutralization of reporter viruses that retained immature structures compared to those grown in the presence of exogenous furin [52]. 

The risk of ADE is one of the primary concerns in DENV vaccine development and was emphasized when young children vaccinated with CYD-TVD had an increased risk of hospitalization upon subsequent dengue infection [53]. In our in vitro ADE experiments, we observed that all of the bDENV-2 sera enhanced DENV infection in K562 cells to comparable degrees at high serum concentrations. However, at subneutralising concentrations of sera, mature bDENV-2 sera had lower levels of ADE, which is consistent with the absence of ADE-inducing antibodies. Newer vaccine candidates such as DSV4, a subunit vaccine shown to minimize the induction of ADE in vivo, have shown comparable in vitro ADE in K562 cells to tetravalent live attenuated vaccine candidates [54]. These discrepancies between in vitro and in vivo enhancement shows the potential drawbacks of the K562 assay system, which might not fully reflect the complexities of ADE in vivo. A possible explanation to these discrepancies is that K562 cells, in contrast to in vivo ADE models, only assess the extrinsic ADE of DENV. K562 cells do not produce the type I interferon and are therefore not suitable for the analysis of intrinsic ADE [55]. Enhancement assays in cell types, such as in primary macrophages [55,56], which can be used to measure both Fc-mediated entry and immune modulation, would be of interest in subsequent studies.

Generating balanced tetravalent immune responses in live-attenuated and inactivated vaccine candidates has been challenging due to varying replication kinetics in attenuated viruses and suboptimal epitope presentation [57,58,59]. The structural authenticity of our bDENV-2 vaccine has been recently reported by our group, and we have shown that it is structurally identical to the DENV-2 virion [39]. The current study provides further validation of the BinJV chimeric vaccine platform for flaviviruses. Previous work by our group and collaborators have shown complete protection in WNV, YFV, ZIKV, and DENV-2 murine challenge models upon immunization with BinJV-based chimeric vaccines [35,37,59,60]. We add to this body of work by showing that independent of virion maturation, two 1 μg intradermal doses of bDENV-2 are 80–100% protective against challenge with DENV-2 in AG129 mice. Of note is the protection conferred by immature particles. Despite lower serum neutralization titers and a higher propensity of ADE in vitro, the vaccination of AG129 mice with immature bDENV-2 conferred 100% protection to virulent DENV-2 challenge. This suggests that protection from homotypic challenge in mice is not dependent on the presentation of epitopes that are specific to the mature virion. Further investigation of the role of maturation in heterosubtypic DENV challenge and analysis of in vivo ADE will provide further clarity for next generation DENV vaccine design strategies.

## Data Availability

The data presented in this study are available within the article and within the Supplementary Materials.

## Supplementary Materials

The following are available online at https://www.mdpi.com/article/10.3390/v13091843/s1. Figure S1: Confirmation of virus recovery and mutagenesis, Figure S2: ADE of DENV 1-4 by mAb 4G2, Table S1: Antigenic analysis of bDENV-2.
