# Supplementary material for: Implications of Dengue Virus Maturation on Vaccine Induced Humoral Immunity in Mice
Source: Viruses. 2021 Sep 15;13(9):1843. doi: 10.3390/v13091843 (PMC8473161; doi:10.3390/v13091843)
Supplement: Supplementary file 1 [file viruses-13-01843-s001.zip › viruses-1299703-supplementary.pdf]

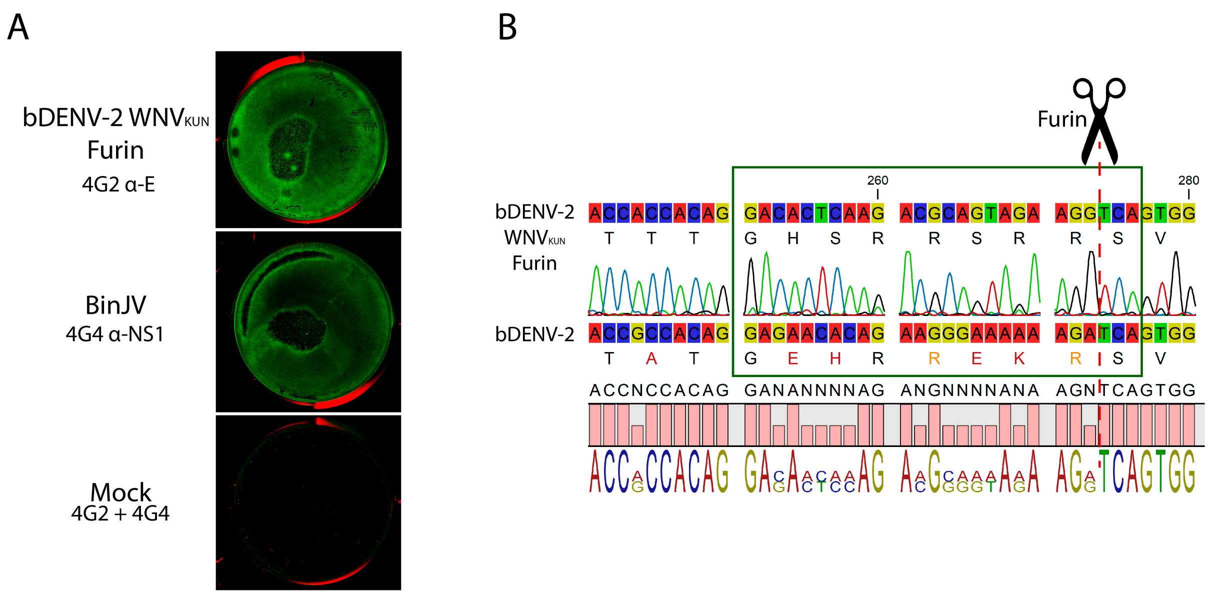

Figure S1. Confirmation of virus recovery and mutagenesis. (**A**) Immunofluorescence staining of fixed C6/36 cells in 6 wells plates. bDENV-2 WNVKUN Furin with α-E mAb 4G2, BinJV control with α-NS1 mAb 4G4, and mock infected with both was detected with goat-anti human IR800. (**B**) Alignment of raw chromatogram from sanger sequencing of bDENV-2 WNVKUN Furin with bDENV-2. Furin cleavage site is indicated by dotted red line and scissors.

Table S1. Antigenic analysis of bDENV-2. Dissociation constant (K_d) and Bmax values from ELISA binding curves for 4G2, C8, 513 and DV62.5 against mature, partially mature and immature bDENV-2.

**mAb**	**Dissociation Constant (K_d)** (nM)	**Bmax** (O.D 450nm)
	Mature bDENV-2	
**4G2**	0.102 ± 0.05	1.774 ± 0.12
**C8**	0.499 ± 0.09	3.029 ± 0.09
**513**	0.223 ± 0.02	2.162 ± 0.04
**DV62.5**	3.002 ± 0.70	0.760 ± 0.04
	Partially Mature bDENV-2	
**4G2**	0.050 ± 0.01	1.613 ± 0.05
**C8**	1.445 ± 0.09	2.793 ± 0.04
**513**	0.162 ± 0.02	2.711 ± 0.06
**DV62.5**	1.045 ± 0.05	1.554 ± 0.01
	Immature bDENV-2	
**4G2**	0.035 ± 0.01	1.760 ± 0.07
**C8**	5.680 ± 1.01	2.239 ± 0.09
**513**	0.240 ± 0.03	3.102 ± 0.04
**DV62.5**	2.686 ± 0.11	2.831 ± 0.026

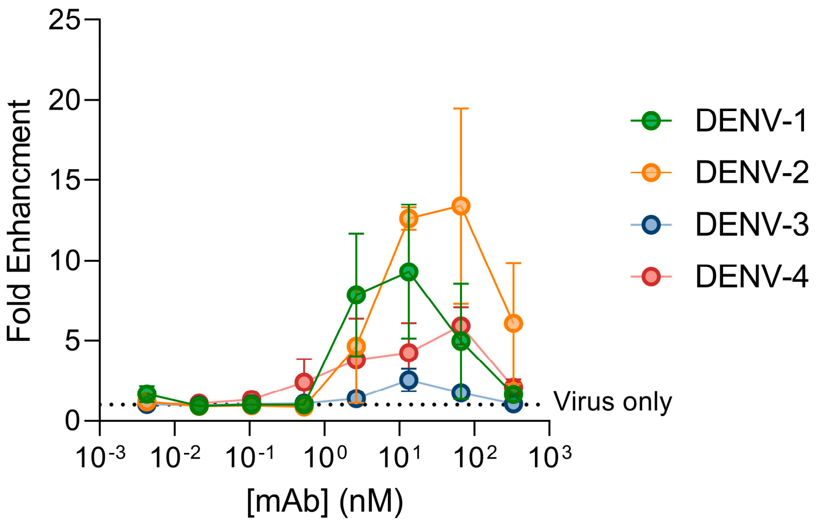

Figure S2. ADE of DENV 1-4 by mAb 4G2. Fold enhancement of infection by 4G2 in K562 cells relative to virus only control of DENV-1, DENV-2, DENV-3, and DENV-4 as determined by sNS1 capture ELISA. Each data point represents the mean of two independent replicates ± SD.
